# Post-Mortem Examinations

**Published:** 1914-02-21

**Authors:** 


### POST-MORTEM EXAMINATIONS.

Once again a sensation-hunting weekly newspaper has been devoting attention to the practice of autopsy in public institutions. The contention implied by the editor is that autopsies are only defensible when there is serious doubt as to the cause of death or under warrant from a coroner. A further innuendo of a less amiable type suggests that autopsies are a way of securing "subjects for students' experiments," whatever that may mean.

The whole tone of this editor's comments reveals a real or pretended ignorance of certain very elementary facts which can be briefly expounded. There is nothing morally wrong, much less against the law, in conducting an autopsy when the sanction of the deceased person's relatives or representatives has been obtained. This applies to all medical men, whether institutional officers or private practitioners. There is no possibility of students or anyone else making "experiments" in the *post-mortem* chamber of an institution. It is true that in all front-rank institutions, whether they are teaching centres or not, permission to conduct an autopsy is almost always asked for by the medical staff. The reason is that the findings of the *post-mortem* chamber are and have always been a fruitful source of new light upon disease, and the starting-point of valuable researches whose aim and end is progress in the fight against disease. In all up-to-date hospitals and infirmaries *post-mortem* examinations are conducted by experts; and the greatest care is taken that nothing shall be done to mutilate the dead beyond the barest minimum, unnoticeable afterwards unless the body is stripped.

To speak of "dissections," "scandals," "revelations," "experiments," and so forth, by way of discrediting the common and necessary *post-mortem* examinations as they are known in hospitals and infirmaries, is merely to create an atmosphere of prejudice and cant about a topic much better not discussed in lay papers. When it is added that the whole structure of misrepresentation is based on one alleged case of autopsy against the wish of a parent, in a case where such investigation was clearly most desirable and is stated to have been desired by a Coroner, it will be seen how mountains can still be made out of mole-hills by those whose sense of responsibility is atrophied. We are just as anxious as anyone else can be that no unsanctioned autopsy shall take place in institutions; but we prefer to help rather than hinder the proportion of such sanctions.

